# Climate Penalty on Air Pollution Abated by Anthropogenic Emission Reductions in the United States

**DOI:** 10.21203/rs.3.rs-3245771/v1

**Published:** 2023-08-16

**Authors:** Lifei Yin, Bin Bai, Bingqing Zhang, Qiao Zhu, Qian Di, Weeberb J. Requia, Joel D. Schwartz, Liuhua Shi, Pengfei Liu

**Affiliations:** 1 School of Earth and Atmospheric Sciences, Georgia Institute of Technology, Atlanta, GA 30332, USA; 2 Gangarosa Department of Environmental Health, Rollins School of Public Health, Emory University, Atlanta, GA 30322, USA; 3 Vanke School of Public Health, Tsinghua University, Beijing, China; 4 School of Public Policy and Government, Fundação Getúlio Vargas, Distrito Federal, Brazil; 5 Department of Environmental Health, Harvard T.H. Chan School of Public Health, Boston, MA 02115, USA

## Abstract

Climate change poses direct and indirect threats to public health, including exacerbating air pollution. However, how a warmer temperature deteriorates air quality, known as the “climate penalty” effect, remains highly uncertain in the United States, particularly under rapid reduction in anthropogenic emissions. Here we examined the sensitivity of surface-level fine particulate matter (PM_2.5_) and ozone (O_3_) to summer temperature anomalies in the contiguous US and their decadal changes using high-resolution datasets generated by machine learning models. Our findings demonstrate that, in the eastern US, efficient emission control strategies have significantly reduced the climate penalty effects on PM_2.5_ and O_3_, lowering the associated population exposure. In contrast, summer and annual PM_2.5_ in the western US became more sensitive to temperature, highlighting the urgent need for the management and mitigation of worsening wildfires. Our results have important implications for air quality management and risk assessments of future climate change.

Climate change is one of the greatest global challenges in the 21^st^ century, exerting adverse impacts on human health^1^. Epidemiological evidence indicates that a rise of 1 °C in summer mean temperature corresponds to an estimated increase in mortality of 1% to 2.5% among older adults in US populations, depending on the climate regions^2,3^. Previous studies have proposed numerous pathways for the health impact of climate change and have evaluated the health burden associated with rising temperature^4–7^. One potential pathway is through interactions with air pollution, as a rising temperature can worsen air quality even without changes in anthropogenic activities, known as the “climate penalty” on air quality^8^. Air pollution alone is also a leading risk factor for human health in many countries. Globally, about 7 million premature deaths per year can be attributed to ambient and household air pollution, and this global health impact has been increasing in the recent decade^9^.

Surface O_3_ and PM_2.5_ are two major air pollutants of greatest health concern, and previous studies have suggested that these pollutants are positively correlated with temperature^8,10–17^. These positive correlations indicate that air quality can serve as a mediator in the overall adverse health effect of climate change by increasing human exposure to air pollutants in a warming climate. Therefore, it is important to consider this pathway in both climate and air pollution management and policy making. Long-term ground measurements are commonly used to examine the relationship between surface concentrations of air pollutants (such as O_3_ and PM_2.5_) and temperature^11,12,18–21^. Although this method can provide the “ground truth” temperature-air pollution relationships based on direct observations, the sparse site distributions and data incompleteness limit our ability to obtain a comprehensive understanding. Chemical transport models, which incorporate the chemistry driving O_3_ and secondary PM_2.5_ formation, have been used to derive the O_3_/PM_2.5_-temperature relationship based on long-term simulations or temperature perturbation simulations^22–24^, providing wide geographical coverage of climate penalty estimates and future projections. Nevertheless, it remains challenging for current chemical transport models to accurately capture the magnitude or even the sign of observed associations between air pollution levels and temperature. This issue is particularly substantial for PM_2.5_ due to the diverse and even opposing responses of different PM species to temperature changes^10^. Box models have also been used to study the influence of temperature on ozone concentration and the related chemical mechnisms^25,26^. This method is difficult to apply to PM_2.5_ because of the spatial variability of composition and thus the response to temperature. Comprehensive and reliable quantification of the sensitivities of O_3_ and PM_2.5_ to temperature, i.e., the climate penalty effect, and how these sensitivities change over time, is essential for predicting future air quality, assessing associated health burdens, and developing relevant public policy.

In this study, by leveraging high-resolution air pollution datasets derived from machine learning, we examined the relationships between summer (June, July, and August; JJA) mean temperature and summer/annual PM_2.5_ (PM_2.5, JJA_/PM_2.5, ANN_) and summer O_3_ (O_3, JJA_) across the contiguous US (CONUS) (see Methods). We utilized 1 km × 1 km gridded surface daily mean PM_2.5_, 8-h maximum O_3_, and surface temperature data over the CONUS from 2000 to 2016 to examine the climate penalty effects. The gridded surface concentration of PM_2.5_ and O_3_ are generated by an ensemble machine learning (ML) model trained by ground observations with land use, meteorology, chemical transport models, and satellite observations as predictors^27,28^. The cross-validation of ML predictions against held-out ground observations has shown good consistency, and the ML-modeled results can fill in the spatial and temporal gaps of sparse ground observations. Nationwide long-term ground observations of major components of PM_2.5_ were used to examine the possible mechanisms.

The obtained historic air pollution-temperature sensitivity datasets in the CONUS can successfully reproduce the ground-based observations while providing hyperlocal spatial resolution and complete spatial coverage, thus improving human exposure assessment. Our results underscore a significant decrease in air pollution-temperature sensitivity and associated health risks in the Eastern US due to anthropogenic emission reductions over the studied period, while a high climate penalty on air pollution remains a large issue in the Western US due to the worsening wildfires. These results imply that reducing the emissions of air pollutants and their precursors can be an effective way to mitigate the potential adverse health impacts of climate change.

## Worsened PM_2.5_ and O_3_ levels in warmer summers

**Figure 1.**
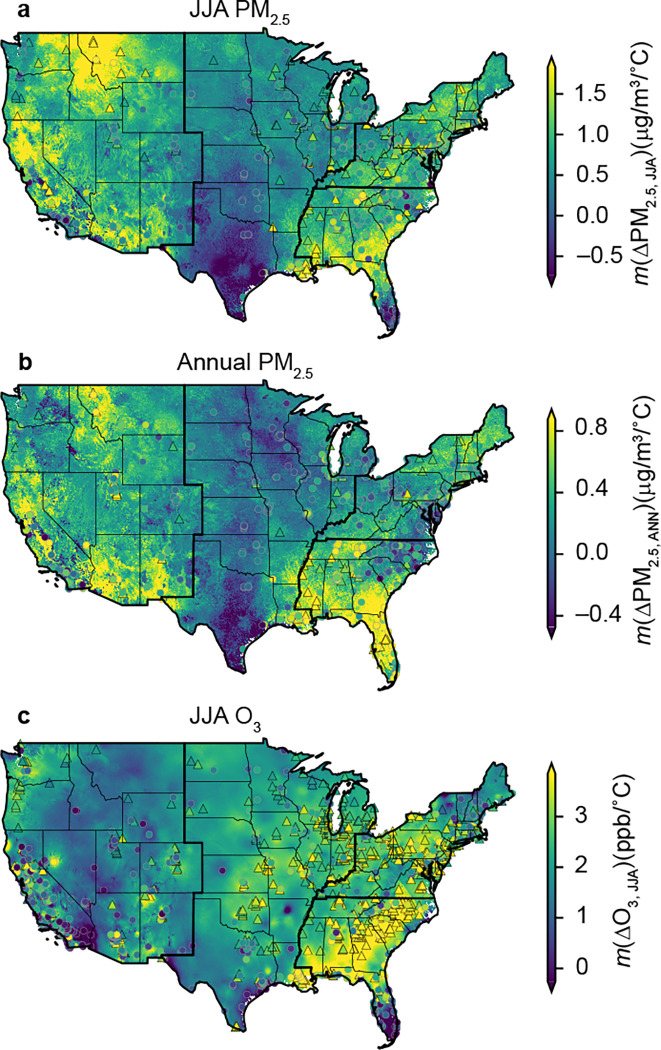
Maps showing the sensitivities of surface air pollutants concentration to anomalies of summer mean temperature derived from high-resolution datasets. Results derived from ground-based measurements (scatters) are also shown for comparison. All results are based on data from 2000–2016. a-c, Temperature sensitivity of summertime PM_2.5_ (a), annual PM_2.5_ (b), and summertime O_3_ (c).

To quantify the relationships between air pollution and summer temperature, the climate penalty effects herein were defined as the slope of detrended air pollution levels versus summer temperature anomalies (see Methods). This method accounts for anthropogenic emission changes over time and variations in air pollution baselines across different locations^8,10,12^. The high-resolution temperature sensitivity map derived from 17-year (2000–2016) ML-modeled data successfully captured the sign, magnitude, and spatial pattern of observed temperature sensitivity of PM_2.5_ and O_3_, providing an estimate of climate penalty effects over the CONUS (Fig. 1). PM_2.5, JJA_ was positively correlated with temperature in most regions, with hotspots in the Western and the Southeastern US (Fig. 1 a). A similar spatial distribution can be found in *m*(ΔPM_2.5,_ ANN), but with generally smaller magnitudes relative to PM_2.5, JJA_ (Fig. 1 b). Positive correlations between summer O_3_ and temperature were ubiquitous, peaking in the Southeastern US (Fig. 1 c). In Texas and southern Florida, sensitivities of PM_2.5_ and its components (Supplementary Fig. S1) were negative, which might be related to the dependence of other meteorological factors (relative humidity, precipitation, wind speed, boundary layer height, etc.) on temperature^19^. Considering that most modeled and observed negative sensitivities are not statistically significant (*p*<0.05) (Supplementary Fig. S1 and Fig. S2), we treated these negative values as no climate penalty effects (insensitive to temperature) in further analysis. The high-resolution sensitivity data demonstrated that the climate penalty effects on summer and annual PM_2.5_ were widespread in the CONUS, while their magnitudes varied considerably across locations.

**Figure 2.**
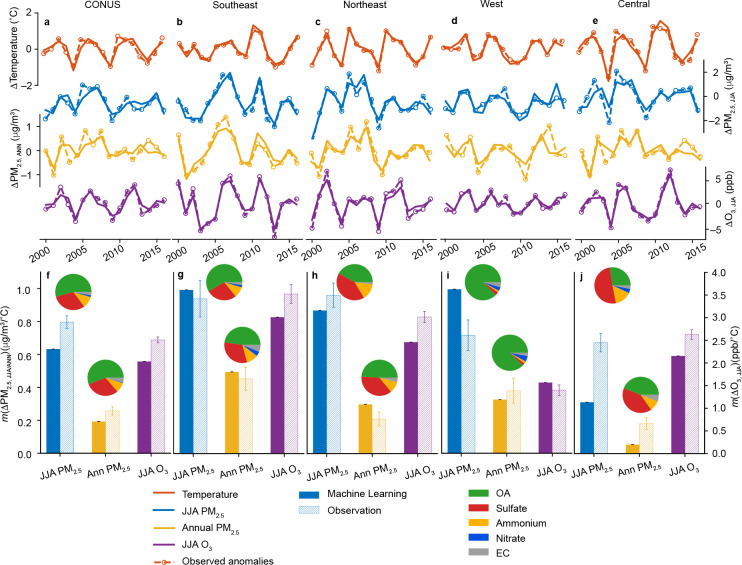
Time series and regression coefficients showing positive correlations between air pollutants and summer temperature for all studied regions. All results are based on data from 2000–2016. a-e, Interannual variation of detrended summer temperature anomalies, summertime PM_2.5_ anomalies, annual PM_2.5_ anomalies, and summertime O_3_ anomalies in the contiguous US (a), Southeastern US (b), Northeastern US (c), Western US (d), and Central US (e); f-j, ML modeled and ground-based observed regional responses of summertime PM_2.5_ (JJA PM_2.5_), annual PM_2.5_ (Ann PM_2.5_), and summertime O_3_ (JJA O_3_) to summer temperature in the contiguous US (f), Southeastern US (g), Northeastern US (h), Western US (i), and Central US (j). Inset pie charts show the contributions of five PM components (organic aerosol (OA), sulfate, ammonium, nitrate, and elemental carbon (EC)) to the overall summertime and annual PM_2.5_-temperature sensitivities.

The regionally aggregated time series indicate that the detrended anomalies of PM_2.5, JJA_, PM_2.5, ANN_, and O_3, JJA_ were positively correlated with summer temperature anomalies in all regions for both ML estimates and ground observations (Fig. 2 a–e). Consistent with the spatial distribution shown in Fig. 1, the largest regional *m*(ΔPM_2.5, JJA/ANN_) was found in the Southeastern and Western US, where a 1 °C increase in temperature was associated with 1 μg/m^3^ increase in summer PM_2.5_ and over 0.3 μg/m^3^ increase in annual PM_2.5_ concentration, respectively (Fig. 2f–j). Notably, the temperature sensitivity of OA was a major source of the overall *m*(ΔPM_2.5, JJA/ANN_), especially in the Western US, where OA alone contributed to ~90% of *m*(ΔPM_2.5, JJA/ANN_). Such a high temperature response of OA level in the Western US was attributable to the primary organic aerosol (POA) from wildfire emissions, which can be highly sensitive to summer temperature anomalies^29,30^. In contrast, the high temperature sensitivity of OA in the Southeastern US was mainly driven by the increased secondary organic aerosol (SOA) formation due to higher biogenic volatile organic compounds (BVOC) emissions and higher aqueous SOA production rates in a warmer summer^10^, contributing 54.5% to *m*(ΔPM_2.5, JJA_). This may also account for the large *m*(ΔPM_2.5, JJA/ANN_) values in the Appalachian zone. Sulfate explains a large fraction of PM_2.5_-temperature sensitivity in the Central US, accounting for 50.7% of *m*(ΔPM_2.5, JJA_). The contribution from sulfate (49.1%) was comparable to that from OA (41.6%) in the Northeastern US. The high sulfate sensitivity to summer temperature can be attributed to the accelerated oxidation rate of sulfur dioxide under higher temperatures, as well as increased sulfur dioxide emissions from the energy generation sectors for a warmer summer^10^.

The climate penalty on O_3_ was remarkably high in the Southeastern US, where 1 °C rise in temperature led to a 3 ppb increase in the summer mean of 8-h maximum O_3_ level. Previous studies proposed several pathways to increase surface O_3_ under a warmer climate, such as enhanced BVOC and soil NO_x_ emissions, accelerated photochemical reaction rates, and facilitated thermal decomposition of peroxyacyl nitrates (PANs)^12,20,24^. Based on chemical transport modeling, previous studies showed a negative correlation between O_3_ and temperature in the Southeastern US and suggested that enhanced BVOC emissions act to decrease O_3_ in this VOC-abundant area^8,24^. However, our results using empirical data suggest a strong positive O_3_-temperature correlation, consistent with the observational evidence in previous studies^12^. The errors of models to reproduce signs and magnitudes of the pollution-temperature relationship would propagate into uncertainties in the predictions of air quality under future climate scenarios.

## Impacts of anthropogenic emission reductions on climate penalty

**Figure 3.**
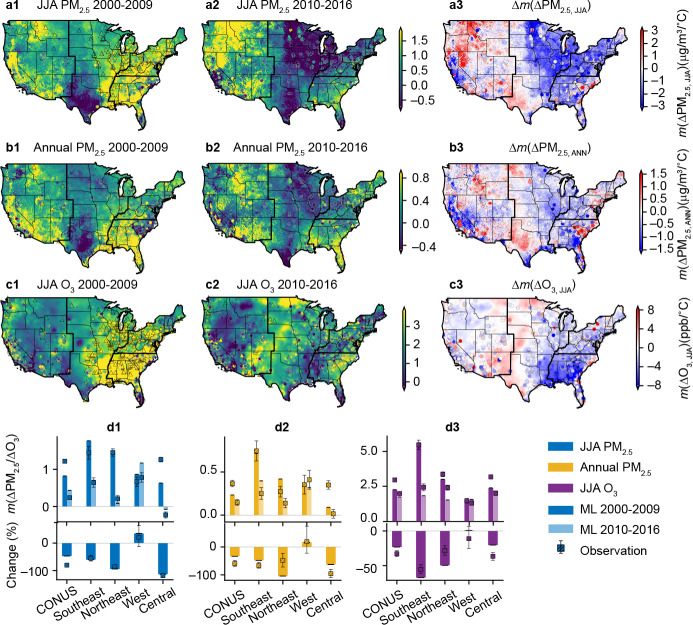
Changes in the spatial patterns and regional mean values of the climate penalty effects from 2000–2009 to 2010–2016. a-c, ML modeled (colored maps) and observed (scatters) temperature sensitivities in 2000–2009, 2010–2016, and the difference between two periods for summertime PM_2.5_ (a1, a2, a3), annual PM_2.5_ (b1, b2, b3), and summertime O_3_ (c1, c2, c3). d, comparisons of regional sensitivities in 2000–2009 and 2010–2016 (upper panels) and relative changes in percentage (lower panels, bars represent the mean percentage changes; error bars represent 95% confidence interval) for summertime PM_2.5_ (d1), annual PM_2.5_ (d2), and summertime O_3_ (d3).

Over the past decade, the climate penalty on air quality decreased considerably in most regions due to reductions in precursor emissions from anthropogenic sources. From 2000–2009 to 2010–2016, large decreases in *m*(ΔPM_2.5, JJA_) were seen in the Eastern US and California, where the air quality is more affected by anthropogenic activities compared with biogenic sources (Fig. 3 a1–a3). Notably, no penalty effect on PM_2.5, JJA_ was found in the Central US after 2009, with *m*(ΔPM_2.5, JJA_) decreasing to negative. Similar spatial patterns of the decadal change can also be seen in *m*(ΔPM_2.5, ANN_) (Fig. 3 b1–b3). The differences in aggregated regional sensitivities in the two periods were significant (Fig. 3 d1–d2). The primary driver of the reduction in PM_2.5_ sensitivity in the Eastern US was the decrease in the temperature sensitivity of sulfate and associated ammonium (Supplementary Fig. S3). During 2000–2009, the temperature sensitivities for inorganic sulfate + ammonium and OA were comparable in most regions (except Western US), while the sensitivities for inorganics were significantly diminished (<0 μg/m^3^/°C) during 2010–2016. Since 2009, the cross-state air pollution rule resulted in a substantial reduction in SO_2_ emissions in the Eastern US. This result shows that the climate penalty on inorganic components of PM_2.5_ was more sensitive to changes in anthropogenic emissions and thus can be effectively mitigated by emission control. Reduced sulfate sensitivity left OA the dominant source of PM_2.5_-temperature sensitivity. The temperature sensitivity of OA remained constant in the Southeastern US, in contrast to the more than 80% decrease in the Northeastern and Central US. A plausible explanation is that biogenic VOCs are major precursors of SOA in the Southeastern US. Therefore, OA sensitivity in the Southeastern US was less sensitive to the reduction of anthropogenic emissions and remained high in 2010–2016^31^. In addition, the prescribed fire emissions, which are not related to anthropogenic emission changes, may also make an important contribution to the OA level in the Southeast, although its contribution to temperature sensitivity is unclear. Meanwhile, anthropogenic VOCs and primary OA contribute most to the OA in the Northeastern and Central US and the OA temperature sensitivity reduced over the studied period in response to the emission control^32,33^.

The O_3_-temperature sensitivity decreased by over 60% in the Southeastern US, much higher than in other regions (Fig. 3 c1–c3). Positive relationships between O_3_ and summer temperature were ubiquitous across the Southeastern US during 2000–2009, with a regional temperature sensitivity two times higher than that in other regions (Fig. 3 d3). The summer mean 8-h maximum O_3_ concentration in this region decreased by 14.4% from 2000–2009 to 2010–2016 because of NO_x_ emission reductions. Over the same period, the regional temperature sensitivity of O_3_ decreased from 5.6 ppb/°C in 2000–2009 (1.6~3 ppb/°C in other regions) to 1.8 ppb/°C in 2010–2016 (1.5~1.9 ppb/°C in other regions). Measurements from ground site observations also confirmed this trend. As NO_x_ emission decreased in the Southeast, O_3_ formation became less sensitive to BVOC emissions, leading to a reduction in *m*(ΔO_3, JJA_).

Notably, the impacts of emission control are more significant in urban areas, which reduced the urban-nonurban differences in climate penalty. During 2000–2009, the increase in O_3_ concentration induced by temperature rise was higher in urban areas (3.3 ppb/°C in CONUS) than in the nonurban areas (2.4 ppb/°C in CONUS), raising the health impacts of the climate penalty due to the large urban population exposed (Supplementary Fig. S4). The production of O_3_ in urban areas is typically more towards the VOC-limited regime due to abundant NO_x_ emissions from traffic and thus is more sensitive to the enhanced BVOC emissions at high temperatures than O_3_ formation in nonurban areas. As the NO_x_ emission decreased, the O_3_ production gradually shifted towards the NO_x_-limited regime less sensitive to BVOC emissions. This shift is more pronounced in urban areas where NO_x_ emissions change more drastically^34^. Due to the decreased sensitivity of O_3_ formation to BVOC emissions, from 2000–2009 to 2010–2016, *m*(ΔO_3, JJA_) declined by 43.3% in urban areas and 27.8% in nonurban areas, leading to a reduced urban-nonurban difference in *m*(ΔO_3, JJA_), particularly in the Northeastern and Central US. The smaller urban-nonurban differences can reduce potential disparities in population exposure to climate penalty on O_3_.

As opposed to the decreasing sensitivity in the Eastern US, the climate penalty effects in the Western US showed an increasing trend. For example, the climate penalty on PM_2.5, JJA_ increased from 0.9 (before 2010) to 1.2 μg/m^3^/°C (after 2010) in the West. Although no statistically significant difference can be seen from the sparse site measurements, the ML data with more comprehensive coverage demonstrated significant increases in regional *m*(ΔPM_2.5, JJA_) and *m*(ΔPM_2.5, ANN_) (Fig 3. d1–d2). In the Northwestern US, where wildfire emissions are the major source of air pollution, a warmer summer can lead to higher PM_2.5_ levels for both summer and annual averages. The increase in OA-temperature sensitivity drives this sensitivity (Supplementary Fig. S3), meaning that 1 °C rise in summer temperature was accompanied by a larger increase in POA concentration from wildfire in 2010–2016 compared to 2000–2009. This result is consistent with previous studies suggesting an increasing trend in the intensity and burned area of wildfire events^29^. This increasing trend in fire risks threatens the local ecosystem and public health. The *m*(ΔO_3, JJA_) also increased in some areas in the Western and Central US. The decrease in climate penalty due to anthropogenic emission reductions did not fully compensate for the increase associated with worsened wildfires in the Western US. In addition, pollutants in fire smoke can be transported for a long distance, affecting air quality in other areas of the country episodically^35^.

## Implications for the US population exposure

**Figure 4.**
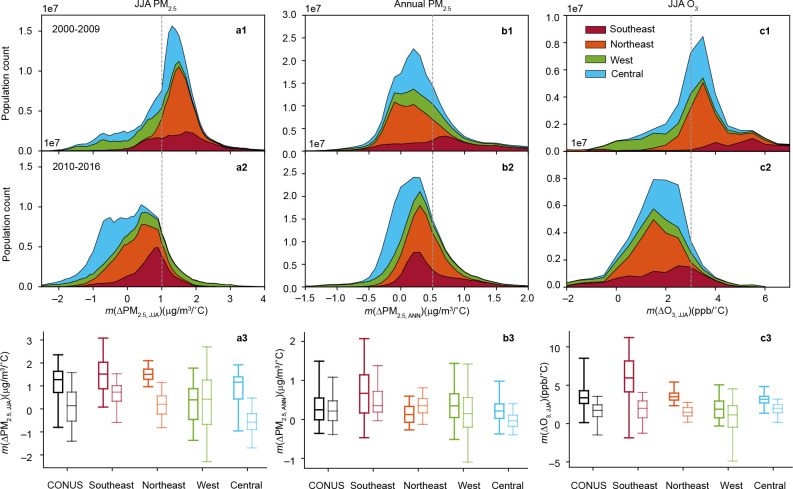
Number and regional distribution of population exposed to air pollution changes associated with 1 °C change in summer temperature for 2000–2009 and 2010–2016. a-c, Stacked area plots showing the fraction of population living in areas with different levels of temperature sensitivity of summertime PM_2.5_ (a), annual PM_2.5_ (b), and summertime O_3_ (c) during 2000–2009 (a1, b1, c1), 2010–2016 (a2, b2, c2), and box plots showing the temperature sensitivity to which 5%, 25%, 50%, 75%, and 95% of the population is exposed, with light colors represent values in 2010–2016 (a3, b3, c3).

The high-resolution “climate penalty” maps allow us to assess the human exposure and vulnerability of the US population to a warmer climate. Combined with high-resolution gridded population data (see Methods), we found that the area where air pollution is highly sensitive to summer temperature decreased substantially over the studied period, thereby reducing population exposure to high climate penalty effects (Fig. 4). Decreases can be found at all exposure levels, with the most significant changes occurring in 75% and 95% of the population exposure levels in most regions (Fig. 4 a3–c3). To assess the changes in exposure to high climate penalty, we used the temperature sensitivities (calculated based on data from 2000–2016) to which 75% of the population is exposed as a threshold representing high climate penalty effects (see Methods).

Specifically, the fraction of area and population of the CONUS under *m*(ΔPM_2.5, JJA_) ≥ 1 μg/m^3^/°C decreased by 30.1% and 51.2% from 2000–2009 to 2010–2016, respectively (Fig. 4 a1–a2; Supplementary Fig. S5 a1-a2; Table S1 and Table S2). Such low shifts were pronounced in all regions except for the Western US. In contrast, the fraction of the population under *m*(ΔPM_2.5, JJA_) ≥ 1 μg/m^3^/°C increased by 11.1% in the Western US owing to the increased *m*(ΔPM_2.5, JJA_) in fire-prone areas. However, the reduction in climate penalty in densely populated regions, such as the Southeastern and the Northeastern US, offset the increased risk in the Western US and led to an overall reduced risk at the national level. The population distribution shifted to a lower climate penalty on O_3_ as a result of overall decreases in *m*(ΔO_3, JJA_) in areas with high population density (Fig. 4 c1–c3). The CONUS population under *m*(ΔO_3, JJA_) ≥ 3 ppb/°C decreased by 82.6% from 2000–2009 to 2010–2016. This improvement was most significant in the Southeast and the Northeast, demonstrating the effectiveness of NO_x_ emission control in improving public health in climate change. In the Southeastern US, approximately 10% of its population was exposed to *m*(ΔO_3, JJA_) as high as 7 ppb/°C during 2000–2009. In the period of 2010–2016, 95% of people in this region experienced *m*(ΔO_3, JJA_) no higher than 4 ppb/°C. The elimination of extremely high O_3_-temperature sensitivity in populous regions may drive a substantial reduction in the health burden associated with climate change. Reduction in exposure to *m*(ΔPM_2.5, ANN_) was less drastic, with a 4.8% decrease in the fraction of the population under *m*(ΔPM_2.5, ANN_)≥0.5 μg/m^3^/°C. Our results indicated that anthropogenic emission reductions have successfully reduced the temperature sensitivity of air pollution in densely populated areas, thereby reducing the vulnerability of climate change associated with air pollution.

Despite the overall reduced population exposure across the CONUS, two threats to public health and exposure disparity remained. First, the temperature sensitivity of OA is becoming a primary driver of the climate penalty on air pollution. In the period of 2010–2016, people in the Southeastern and Western US constituted 80.6% (29.1% in 2000–2009) of the total US population living in areas with high climate penalties on summertime PM_2.5_ (Supplementary Table S1). To reduce OA sensitivity, wildfire management seems to be essential in the Western US. In the Southeastern US, the enhanced BVOC level may be the main driver for the temperature sensitivity of SOA formation. Although anthropogenic sulfate and NO_x_ can influence SOA formation in many aspects and the control of anthropogenic emissions reduced the overall OA level^31,33^, the climate penalty on OA remained in this region. However, the significant decrease in OA sensitivity in the Northeastern and Central US indicated that there might be a threshold of BVOC and sulfate/NO_x_ ratio below which the SOA formation would be more sensitive to anthropogenic emission reductions. Furthermore, the climate penalty on annual PM_2.5_ level increased in the Northeastern US, home to over 30% of the US population. In the Northeast, the fraction of population under *m*(ΔPM_2.5, ANN_)≥0.5 μg/m^3^/°C increased by 18.7% due to increased *m*(ΔPM_2.5, ANN_) in some east coast areas (Fig. 4 b1–b3; Supplementary Table S1). As a caveat, the sensitivity of annual OA level, which might be responsible for the increased *m*(ΔPM_2.5, ANN_) in the Northeast, showed large uncertainties (Supplementary Fig. S3 a2-e2).

Second, the vulnerability to climate change has increased in the Western US, particularly for low-income residents near fire-prone regions^36^. The dominant role of wildfires determined that the people in these areas have been exposed to a higher PM_2.5_ level in a warmer summer, and this situation worsened over time. From 2000–2009 to 2010–2016, climate penalty effects on both PM_2.5_ and O_3_ in these areas decreased less, if not increased, than in densely populated areas that benefited from anthropogenic emission reductions (Fig. 3 a3–c3). The larger decrease in more populous areas can also be seen in the large decrease in exposure level for 5% of the population versus the small decrease in exposure level for 5% of the area (Fig. 4 a3–c3; Supplementary Fig. S5 a3-c3). That means high-income populations living in the originally low climate penalty areas (near urban areas on the west coast) will experience a further decline in penalty effects due to anthropogenic emission reductions. In comparison, low-income populations living with the originally high penalty (fire-prone areas) are expected to experience higher penalty effects. Moreover, PM_2.5_ concentration in the west coast areas decreased during the studied period, whereas the concentration in fire-prone areas increased (Supplementary Fig. S6). This phenomenon indicates potential exposure disparities and climate justice, which warrant more attention for future studies.

## Discussion

Understanding the responses of surface air pollution to temperature is crucial for predicting future air quality trends in response to climate change and assessing the associated health burden. By leveraging long-term, high-resolution PM_2.5_ and O_3_ estimates derived from machine learning models, we analyze air quality responses to summer temperature throughout the contiguous US. The machine learning datasets not only successfully reproduced the temperature sensitivities of PM_2.5_ and O_3_ observed at ground stations but also provided more comprehensive spatial coverage and thus better representations of regional sensitivities and human exposure estimates. The generated high-resolution data of historic temperature sensitivities of PM_2.5_ and O_3_ can also be useful for evaluating the performance of chemical transport models.

Our results highlight that the climate penalty effects on PM_2.5_ and O_3_ were historically widespread in the contiguous US. However, the extent of the deterioration of air quality due to warmer summer temperatures has been effectively abated or even eliminated by anthropogenic emission reductions in the Eastern US, suggesting that reduction in anthropogenic emissions can be an efficient way to mitigate the adverse health impacts and vulnerability of climate change. It is worth noting that our results hold true for most months, although increased sensitivity is seen in transition periods (e.g., May and October, Supplementary Fig. S7). Remarkably, the temperature sensitivity of sulfate (and associated ammonium), which is most responsive to emission reduction, has almost vanished during the past decade, leaving the OA a dominant contributor to the temperature sensitivity of PM_2.5_. Therefore, deteriorated PM_2.5_ due to temperature rise remained prominent in the Southeastern and Western US in 2010–2016. The temperature response of biogenic SOA formation is less sensitive to the change of anthropogenic emissions, although anthropogenic sulfate and NO_x_ can also influence SOA formation^31,37^. On the other hand, the strong positive correlation between O_3_ and temperature in the Southeast is highly sensitive to anthropogenic emissions and has been substantially mitigated by reductions in NO_x_ emissions over the studied period. The increasing trend of PM_2.5_-temperature sensitivity in the Western US suggests that wildfire emissions have become more sensitive to temperature anomalies in recent years under rapid climate change. Potential factors contributing to the exacerbated temperature response of wildfire emissions include more fire occurrences, larger burning areas, longer fire durations, etc. The mechanisms underpinning this phenomenon are worth further investigation^29,30^.

There are few studies focusing on the climate penalty on PM_2.5_ due to the different responses of its components^14,21,22^. The detailed analysis of the PM_2.5_-temperature relationship in this study can be valuable for model development and validation. For future air quality projections, correctly capturing the temperature-sensitive processes can be more important than reproducing the observed concentrations^22^. Considering the radiative effects of aerosols, these processes can also be important for modeling climate-aerosol feedback^38^. Although we expect minimized impacts from sulfate aerosol with continually decreasing sulfur dioxide emissions, the sensitivity associated with OA remains significant. Most organic species scatter solar radiation, while there are also light-absorbing components of organic aerosol known as ‘brown carbon’^39^. Therefore, future climate projections need to consider the temperature responses of OA and its radiative effects^40,41^. In this study, we used ground-based measurements to diagnose the temperature sensitivity of PM_2.5_ species to investigate the main drivers of overall PM_2.5_ sensitivity. Due to data availability, species and PM_2.5_ concentrations do not necessarily come from the same site or on the same day, leading to differences in PM_2.5_ changes and changes in summed species concentrations. Future work can benefit from the emerging high-resolution PM_2.5_ species dataset from machine learning models^42^, and more accurate analyses can be performed to improve understanding of the climate penalty on PM_2.5_.

Many processes contribute to the temperature sensitivity of air quality, for instance, precursor emissions, chemical reaction rate, stagnation, etc^10^. Observations and the machine learning data used in our study can only provide information on the total derivative of air pollution and temperature (*d*[O_3_]/*d*T, *d*[PM_2.5_]/*d*T, *d*[OA]/*d*T). Chemical transport models are required to break down the total sensitivity and quantify contributions from the individual processes, i.e., stagnation (∂[PM_2.5_]/∂[stagnation]* ∂[stagnation]/∂[T]), biogenic VOC emissions (∂[OA]/∂[BVOC]* ∂[BVOC]/∂[T]), anthropogenic SO_2_ emissions (∂[sulfate]/∂[SO_2_]* ∂[SO_2_]/∂[T]), etc^43^. However, one prerequisite to understanding the process contribution is that models can reproduce the observed total derivative. In this sense, the high-resolution sensitivity data generated in this study is necessary to evaluate model skills, identify potential sources of model biases, and improve model performance in resolving these present-day relationships, building confidence in mechanism analysis. Understanding individual processes has important implications for future air quality projection. Our results also indicate that the statistical relationships for future projections built upon historic observations need to consider complex interactions of air quality and climate with changing anthropogenic emissions. In addition to temperature, many other meteorological parameters, such as precipitation and wind speed, can also play an important role in such interactions.

## Methods

### High-resolution PM_2.5_ /O_3_ concentration and temperature data.

Ground-level daily mean PM_2.5_ and 8-hour maximum O_3_^28^ across the contiguous United States were generated using an ensemble machine-learning approach in previous studies^27,28^. Briefly, three machine learning algorithms (neural network, random forest, and gradient boosting) were trained based on multiple predictor variables, including satellite data, meteorological variables, land-use variables, elevation, chemical transport model simulations, reanalysis datasets, and others. The PM_2.5_ and O_3_ concentrations were modeled with each algorithm. A generalized additive model was used to combine estimates and obtain an overall prediction. The ensemble model demonstrates good performance with a 10-fold cross-validated R^2^ of 0.86 for daily PM_2.5_ predictions, 0.89 for annual PM_2.5_ predictions, and 0.90 for daily maximum 8 h O_3_. The average RMSE (root mean square error) was 2.79 μg/m^3^ for daily PM_2.5_ and 4.55 ppb for daily maximum 8 h O_3_, respectively. Detailed information on PM_2.5_ and O_3_ datasets can be found in Di et al. (2019)^27^ and Requia et al. (2020)^28^, respectively. The PM_2.5_ data can be accessed at https://sedac.ciesin.columbia.edu/data/set/aqdh-pm2-5-concentrations-contiguous-us-1-km-2000-2016, and O_3_ data can be accessed at https://sedac.ciesin.columbia.edu/data/set/aqdh-o3-concentrations-contiguous-us-1-km-2000-2016.

Both datasets have a spatial resolution of 1 km × 1 km. The temporal resolution is daily. Gridded daily maximum and minimum temperatures are obtained from Daymet Daily Surface Weather Data on a 1 km × 1 km Grid for North America, Version 4 R1 (https://doi.org/10.3334/ORNLDAAC/2129). The mean temperature is calculated by summing the maximum and minimum temperatures and dividing by 2.

### Ground-based observations.

Daily measurements of O_3_, PM_2.5_, and particulate components (sulfate, nitrate, ammonium, organic carbon, and elemental carbon) were obtained from the Air Quality System (AQS) network managed by US Environmental Protection Agency (EPA), The Interagency Monitoring of Protected Visual Environments (IMPROVE), Clean Air Status and Trend Network (CASTNET), and The SouthEastern Aerosol Research and Characterization (SEARCH). A factor of 2.1 was used to convert organic carbon mass concentration to organic aerosol mass concentration^44,45^. The pollutant samples are collected for 24 h every three days at most monitoring sites. Monthly mean concentrations are calculated for months with more than 6 daily measurements. Summer mean concentrations are calculated for the summer with at least two monthly data for July, August, or September. Annual mean concentrations are calculated for the year with more than eight monthly mean data. The sensitivity diagnosis was only performed for monitoring stations with at least 11 years of summer or annual mean measurements during 2000–2016.

Temperature observations are from Global Summary of the Month (GSOM), Version 1, provided by the National Centers for Environmental Information (NCEI, https://www.ncei.noaa.gov/access). NCEI stations within a 30 km radius of the AQS stations are averaged and considered co-located pollutant and temperature measurements. The criteria used to select AQS data for the summer averages calculation and sensitivity examination were also applied to the daily NCDC temperature data processing.

### Gridded population dataset.

The gridded population distribution of the US is from the UN WPP-adjusted population count/density, v4.11 product provided by Gridded Population of the World (GPW), v4 database (https://sedac.ciesin.columbia.edu/data/collection/gpw-v4). Data at the native 30 arc-second (approximately 1 km) resolution are used. The population dataset was matched with the high-resolution air pollution datasets by the nearest central grids. The 2010 and 2020 population count data at 30 arc-second resolution was used to assess changes in population exposure from 2000–2009 to 2010–2016.

### Quantification of air pollution sensitivity to summer temperature.

Following the methodology described by Fu et al.^12^, the sensitivity of air pollution levels to temperature, i.e., the climate penalty, is calculated as the slope of the linear regression line for detrended air pollutant concentration anomalies (e.g., ΔPM_2.5_) and detrended temperature anomalies (ΔT). Interannual anomalies of pollutant concentration and temperature were calculated by removing the long-term means. The linear trend was removed to exclude the influence of concentration decrease due to anthropogenic emission reduction. We focused on the climate penalty effects on O_3_ and PM_2.5_ concentrations at ground level due to their direct impacts on public health.

As shown in supplementary Fig. S7, O_3_ and PM_2.5_ concentrations are positively associated with temperature over the warm season (May-October). In light of the mediation role of air pollution in the increased mortality due to the rise in summer mean temperature^2,46^, as well as the co-occurrence of heatwaves and severe air pollution during the summertime that has been observed in many populous regions around the world^15,47^, we mainly focused on the responses of summer air pollution (PM_2.5, JJA_ and O_3, JJA_) to summer temperature changes in this study. While O_3_ pollution is mainly a warm-season issue due to photochemical reactions, PM_2.5_ is a year-round air quality concern because of the complex seasonal variations^10^. To assess the potential lags or long-term effects of warmer summers on air quality, we also investigated the temperature sensitivity of annual average PM_2.5_ (PM_2.5, ANN_) in the 12 months after summers (from June to May of the following year). The climate penalty on summertime PM_2.5_, annual PM_2.5_, summertime O_3_ were denoted as *m*(ΔPM_2.5, JJA_), *m*(ΔPM_2.5, ANN_), *m*(ΔO_3, JJA_), respectively. The sensitivities derived from the high-resolution dataset are based on data from 17 consecutive years (2000–2016), while the observed sensitivities are based on the 11 or more years of data available at each site.

Different regions in the CONUS were investigated separately to illustrate the spatial heterogeneity of pollutant-temperature relationships. The CONUS was divided into four regions (Supplementary Fig. S8): the Southeastern US, the Northeastern US, the Western US, and the Central US. The regional responses were obtained based on all detrended air pollution and temperature anomalies within each region (Supplementary Fig. S9). We also adopt a bootstrapping approach to calculate regional sensitivity. The slope and standard error based on bootstrapping method were comparable to that based on the linear regression models.

Regional temperature sensitivity derived from ML datasets was comparable to that based on measurements (Fig. 2 f–j). Larger discrepancies were found in the Western and Central US due to lower monitoring station coverage. Therefore, data in grids co-locating with observations (within a 5 km radius) were used to validate the ML datasets. As shown in Supplementary Fig. S10 (compared to Supplementary Fig. S9) and Supplementary Fig. S11 (compared to Fig. 2 a–e), the agreement was improved in all regions when only co-located data were used in the analysis. Considering that the ML approach combined multi-source datasets (ground observation, satellite observation, and chemical transport models) and successfully reproduced not only the observed long-term concentration but also the magnitude and spatial distribution of temperature sensitivity, we assumed that regional mean sensitivity derived from this dataset would be more representative than the sensitivity derived from sparse ground-based observations.

The varied temperature-dependences of its species complicate the PM_2.5_-temperature relationship^21^. The associations between temperature and five principal PM_2.5_ components, including sulfate, nitrate, ammonium, organic aerosol (OA), and elemental carbon (EC) were investigated using the long-term observation derived from AQS database. Sensitivities at each site are shown in Supplementary Fig. S1, and regional sensitivities are shown in Supplementary Fig. S12 It should be noted that *m*(ΔPM_2.5_) is not necessarily equal to the sum of sensitivities of each species because of different locations, numbers, and times of observations used to fit the slopes. The inset pie charts in Fig. 2 (main text) showed species contributions to overall *m*(ΔPM_2.5, JJA_) and *m*(ΔPM_2.5, ANN_). Temperature sensitivity of summer nitrate concentration in the Northeast was negative with a slope of −0.005 μg/m^3^/°C and thus was not included in the pie chart. As shown in Supplementary Fig. S12, the nitrate sensitivity ranges from −0.005 to 0.024 μg/m^3^/°C, making a small contribution to the overall PM_2.5_ sensitivity.

Anthropogenic emissions were substantially reduced during the past decade. As a result, summer PM_2.5_, annual PM_2.5_, and summer O_3_ concentrations show a decreasing trend in most regions (Supplementary Fig. S13). From 2000–2009 to 2010–2016, the summer PM_2.5_, annual PM_2.5_, and summer O_3_ concentrations in CONUS decreased by 23.6%, 30.5%, and 8.9%, respectively. Likewise, the decreasing trend can be found in the temperature sensitivity of air pollution for the thirteen 5-year periods during 2000–2016 (Supplementary Fig. S14). The impacts of anthropogenic emission reductions on climate penalty were further studied by comparing the sensitivities in 2000–2009 and 2010–2016. The regional sensitivities calculated by the bootstrapping method were used to calculate the percentage change and its confidence interval of each region (Fig. 3 d1–d3). The changes in population exposure associated with decreased climate penalty were evaluated by comparing population distribution under different levels of climate penalty effects during 2000–2009 and 2010–2016. The high temperature sensitivities (based on data from 2000–2016) experienced by approximately 25% of the population were selected as the threshold value of high climate penalty effects. The threshold value for *m*(ΔPM_2.5, JJA_), *m*(ΔPM_2.5, ANN_), *m*(ΔO_3, JJA_) are 1 μg/m^3^/°C, 0.5 μg/m^3^/°C, and 3 ppb/°C, respectively. Because of the unevenly distributed population, the area distribution was also studied to show the exposure changes in the US.

The difference in the temperature sensitivity of O_3_ concentration in urban and non-urban areas was examined. The urban/nonurban grids were identified using the GPW population density data in 2010 and 2020. Grids with a population density ⩾ 400 people/km^2^ are considered urban grids^48^.

## Data Availability

High resolution daily mean PM2.5 and 8-h maximum ozone datasets are publicly available at NASA Socioeconomic Data and Applications Center (PM_2.5_: https://sedac.ciesin.columbia.edu/data/set/aqdh-pm2-5-concentrations-contiguous-us-1-km-2000-2016/; O_3_: https://sedac.ciesin.columbia.edu/data/set/aqdh-o3-concentrations-contiguous-us-1-km-2000-2016^49,50^. Gridded temperature data is from Daymet Daily Surface Weather Data on a 1-km Grid for North America, Version 4 R1 (https://doi.org/10.3334/ORNLDAAC/2129)^51^. Temperature observations are from Global Historical Climatology Network - Daily (GHCN-Daily), Version 3, provided by the National Climatic Data Center (NCDC, https://www.ncei.noaa.gov/access). Gridded population distribution of the US is from the UN WPP-adjusted population count/density, v4.11 product provided by Gridded Population of the World (GPW), v4 database (https://sedac.ciesin.columbia.edu/data/collection/gpw-v4)^52^. Other data that support the plots and other findings of this work are available at https://doi.org/10.5281/zenodo.8109660.
